# New Books

**Published:** 2009-06

**Advances in Molecular Toxicology, Vol. 3**

James C. Fishbein, ed.

St. Louis, MO: Elsevier, 2009. 216 pp. ISBN: 978-0-444-53357-9, $235

**Advice on the Department of Energy’s Cleanup Technology Roadmap: Gaps and Bridges**

Committee on Development and Implementation of a Cleanup Technology Roadmap; National Research Council

Washington, DC:National Academies Press, 2009. 280 pp. ISBN: 978-0-309-13231-2, $58.253

**Airborne Radioactive Contamination in Inhabited Areas**

K.G. Andersson

St. Louis, MO:Elsevier, 2009. 250 pp. ISBN: 978-0-08-044989-0, $155

**America’s Energy Future: Technology and Transformation**

Committee on America’s Energy Future, Phase1, National Research Council

Washington, DC:National Academies Press, 2009. 180 pp. ISBN: 978-0-309-11602-2, $40

**Arsenic: Environmental Chemistry, Health Threats and Waste Treatment**

Kevin Henke

Hoboken, NJ:John Wiley & Sons, 2009. 588 pp. ISBN: 978-0-470-02758-5, $190

**Environmental Futures, Vol. 2: The Practice of Environmental Scenario Analysis**

J. Alcamo, ed.

St. Louis, MO:Elsevier, 2009. 212 pp. ISBN: 978-0-444-53293-0, $170

**Environmental Toxicants: Human Exposures and Their Health Effects, 3rd ed.**

Morton Lippmann

Hoboken, NJ:John Wiley & Sons, 2009. 1,167 pp. ISBN: 978-0-471-79335-9, $195

**Fundamentals of Toxicologic Pathology, 2nd ed.**

Wanda M. Haschek, Matthew A. Wallig, Colin G. Rousseaux

St. Louis, MO:Elsevier, 2009. 700 pp. ISBN: 978-0-12-370469-6, $199.95

**Gaia in Turmoil: Climate Change, Biodepletion, and Earth Ethics in an Age of Crisis**

Eileen Crist, H. Bruce Rinker, eds.

Cambridge, MA:MIT Press, 2009. 352 pp. ISBN: 978-0-262-03375-6, $54

**Global Environmental Change and Human Security**

Richard A Matthew, Jon Barnett, Bryan McDonald, Karen L. O’Brien, eds.

Cambridge, MA:MIT Press, 2009. 328 pp. ISBN: 978-0-262-01340-6, $50

**Human and Ecological Risk Assessment: Theory and Practice**

Dennis J. Paustenbach, ed.

Hoboken, NJ: John Wiley & Sons, 2009. 1,592 pp. ISBN: 978-0-470-25319-9, $125

**Human Footprints on the Global Environment: Threats to Sustainability**

Eugene A. Rosa, Andreas Diekmann, Thomas Dietz, Carlo C. Jaeger, eds.

Cambridge, MA:MIT Press, 2009. 328 pp. ISBN: 978-0-262-01315-4, $54

**Learning Science in Informal Environments: People, Places, and Pursuits**

Philip Bell, Bruce Lewenstein, Andrew W. Shouse, Michael A. Feder, eds.

Washington, DC:National Academies Press, 2009. 400 pp. ISBN: 978-0-309-11955-9, $49.95

**Linkages of Sustainability**

Thomas Graedel, Ester van der Voet, eds.

Cambridge, MA:MIT Press, 2009. 430 pp. ISBN: 978-0-262-01358-1, $40

**Modeling Indoor Air Pollution**

Darrell W Pepper, David Carrington

Hackensack, NJ:World Scientific, 2009. 360 pp. ISBN: 978-1-84816-324-9, $98

**Risk Communication: A Handbook for Communicating Environmental, Safety, and Health Risks**

R. Lundgren, Andrea McMakin

Hoboken, NJ: John Wiley & Sons, 2009, 376 pp. ISBN: 978-0-470-41689-1, $79.95

